# Observing a Movement Correction during Walking Affects Evoked Responses but Not Unperturbed Walking

**DOI:** 10.1371/journal.pone.0104981

**Published:** 2014-08-18

**Authors:** Frank Behrendt, Marc H. E. de Lussanet, Heiko Wagner

**Affiliations:** 1 Movement Science, Westf. Wilhelms-Universität Münster, Münster, Germany; 2 Psychology, Westf. Wilhelms-Universität Münster, Münster, Germany; University of Iowa Carver College of Medicine, United States of America

## Abstract

Seeing an action activates neurons in the premotor, motor, and somatosensory cortex. Since a significant fraction of these pyramidal neurons project to the spinal motor circuits, a central question is why we do not automatically perform the actions that we see. Indeed, seeing an action increases both cortical and spinal excitability of consistent motor patterns that correspond to the observed ones. Thus, it is believed that such imitative motor patterns are either suppressed or remain at a sub-threshold level. This would predict, however, that seeing someone make a corrective movement while one is actively involved in the same action should either suppress evoked responses or suppress or modulate the action itself. Here we tested this prediction, and found that seeing someone occasionally stepping over an obstacle while walking on a treadmill did not affect the normal walking pattern at all. However, cutaneously evoked reflexes in the anterior tibial and soleus muscles were modulated as if the subject was stepping over an obstacle. This result thus indicates that spinal activation was not suppressed and was neither at sub-threshold motor resonance. Rather, the spinal modulation from observed stepping reflects an adaptive mechanism for regulating predictive control mechanisms. We conclude that spinal excitability during action observation is not an adverse side-effect of action understanding but reflects adaptive and predictive motor control.

## Introduction

Reflexes are highly adaptive and control movements in a purposeful manner. The adaptability is manifested in task-dependency [1] as well as in its phase-dependency [2]–[5]. This is functionally meaningful and allows reflexes to be integrated into complex movements initiated by supraspinal commands [6]. Moreover, in preparation for an upcoming movement task reflexes can adapt in advance to assist the performance of that task [7], [8]. Reflex gain can even change due to the mere observation of an action [9]–[11] and is modulated in the same manner during passive observation of walking and during active execution of the same action [12].

The concept of motor resonance and its physiological underpinning in the mirror neuron system is well established. This means, that action observation leads to activity of neurons and networks in the brain that are also involved in action execution. Moreover, the activity of individual neurons is known to be similar in observation and execution. Studies suggest that there is an enhanced BOLD response for actions in specialists than in amateurs [13]–[16].

Action observation also modulates the motor responsiveness to Transcranial magnetic stimulation (TMS). Using TMS, a number of studies have shown that motor excitability, measured as motor evoked potentials (MEPs), is modulated due to the mere observation of actions [17], [18], that these changes are specific for the muscles involved in the observed action [19]–[21] and that action execution and action observation are coupled in terms of temporal coding [11], [22], [23]. In addition, it has been reported that the muscular force requirements of observed actions are reflected in TMS-induced motor evoked potentials [24], [25].

A fundamental question that has been around ever since the discovery of mirror neurons is why we do not automatically produce the actions that we see [26]–[28]. Since mirror neurons are pyramidal neurons of the premotor and motor cortex, this is not a trivial problem. It has been proposed initially that the modulation of MEPs during the passive visual observation of actions functions to inhibit the automatic performance of actions that one observes [9]. However, when we evoked reflex responses during the passive viewing of walking we found that the evoked EMG responses were modulated in a dynamic manner, in phase with the visually presented walking cycle. The nature of these dynamically changing modulations was in the same direction as known for active walking so that the modulations during passive viewing cannot help to suppress the automatic copying of observed walking [12]. This finding was confirmed also for TMS stimulation [25], [28], [29]. In the latter study, it was found that the modulation of transcranially evoked motor responses during the visual observation of a reach-grasp-lift-place action sequence changes dynamically with the action and even in a predictive manner [25].

Thus, motor actions are more easily evoked when one is observing and dynamically anticipating the congruent motor action. This dynamic modulation can be measured by transcranial stimulation, suggesting the involvement of high-level cortical mechanisms, but also by the stimulation of the peripheral motor system, suggesting the involvement of lower-level or even spinal motor mechanisms. Consequently, the question as to how automatic mirroring is prevented is still unsolved.

One possible solution to this conundrum is that the suppression of evoked responses is not measured because it is not needed in typical experimental situations. In studies so-far, the observers have typically been stimulated while sitting relaxed in front of a display, a posture in which motor thresholds are high. One might thus argue that evoked responses should be suppressed or even reversed if the observer of the action simultaneously performs a motor act.

Alternatively, mirror properties are confined to neurons that prepare the peripheral motor system for fast postural responses rather than affecting or modifying the ongoing action [12]. If so, we expect that the observation of a motor act does not change ones ongoing motor acts at all. Importantly however, fast responses to unexpected events, such as cutaneous electric stimulation, should be modified and enhanced in a manner that is consistent with the observed action.

To test this hypothesis, we measured the evoked responses while the observers saw the same action. For this, we partially adopted the design of a previous study in which subjects walked on a treadmill while occasionally stepping over an obstacle. On the basis of reflexive responses to cutaneous electric stimulation it was found that stepping over an obstacle is associated with anticipatory spinal activity before the execution of obstacle avoidance steps [8]. In the present study the subjects also walked on a treadmill. However, instead of stepping over an obstace they saw the presented point-light actor stepping over an obstacle. The subjects walked at continuous speed throughout the experiment, while keeping in phase with the point-light actor. Cutaneous electric stimulation was applied to the right ankle just prior to the right-foot stepping movement of the point-light actor.

We used point-light biological motion as visual stimulus, which does not contain image information but can easily be recognized [30]. Motion perception via such point-light displays activates motor- and somatosensory representations in the brain [31]–[34]. These brain regions are thought to fulfill a central role during observation of movement; it is active during action execution as well as during action recognition [31], [35]–[37]. Depending on the task, the observation of point-light biological motion does not only activate these cortical networks but can also modulate spinal reflexes, i.e., the gain of cutaneous reflex in TA [12].

We hypothesized that motor resonance processes at the level of the spinal motor system can also be detected in persons that actively perform a movement themselves. More specifically, we expected that, in walking persons, reflex responses of the *tibialis anterior* muscle (TA) and *soleus* muscle (SOL) would be modulated anticipatory according to visually presented obstacle stepping. We also assumed that the changed reflex gain would not automatically result in a modulation of the actively performed locomotion.

## Materials and Methods

### Participants

Eight healthy, right-handed subjects participated in the study (2 females, 6 males, mean age 31.6±8.6 years). All gave written, informed consent prior to participation, and were informed that they could quit participation at any time. All participants were naive about the scientific purpose of the experiment.

The experiment was approved by the local ethics committee (Örtliche Ethikkomission FB 07 der WWU Münster) and conformed with The Code of Ethics of the World Medical Association (Declaration of Helsinki). Each participant gave written informed consent. No potentially identifying information was stored with the data.

### Visual stimuli

**Figure 1:**
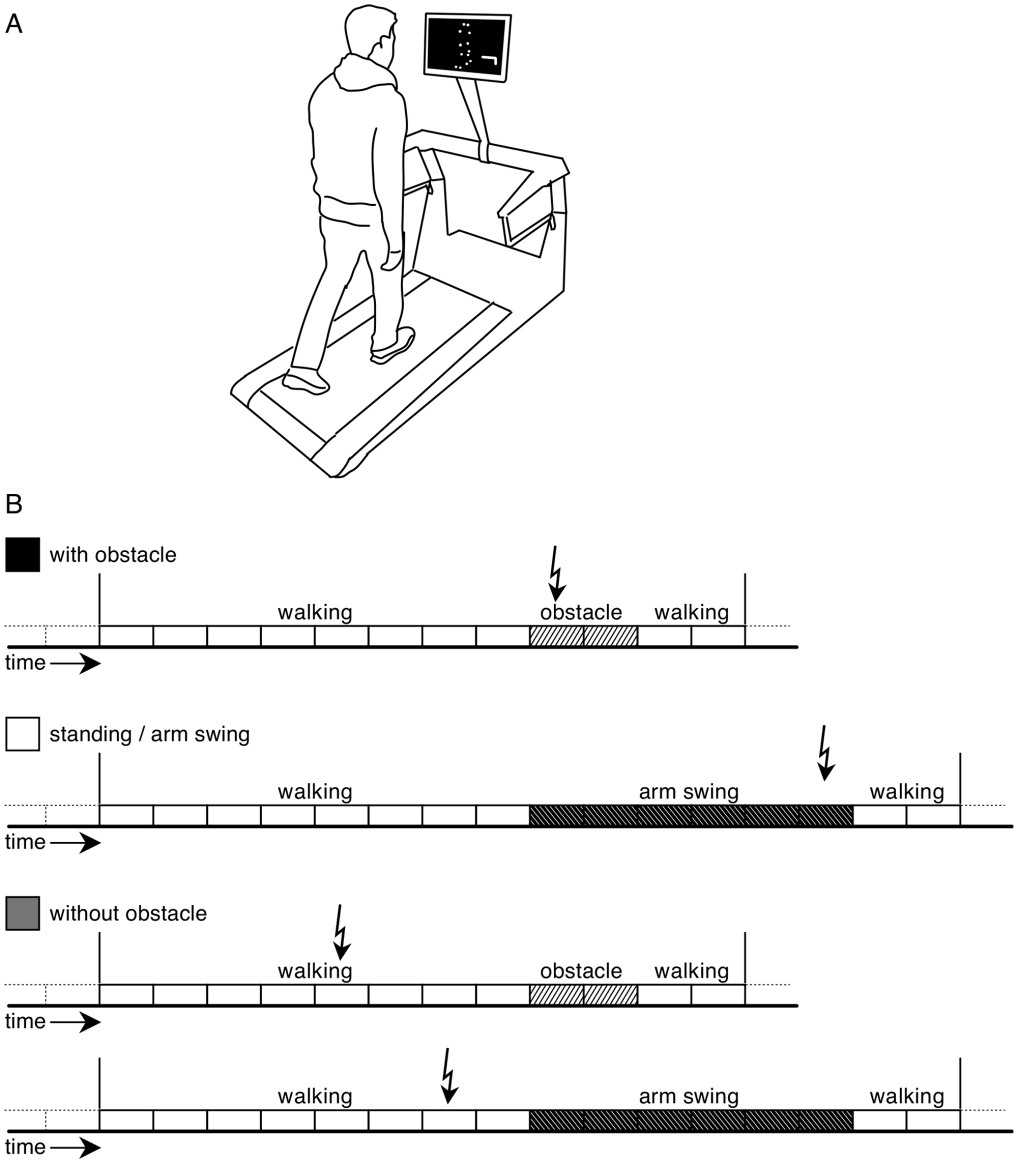
A, Experimental setup illustrating a subject walking on a treadmill while watching a walking point-light stimulus from an oblique back view. Speed of the treadmill was adjusted to match each subjects natural walking frequency with the stimulus. The subjects were asked to walk continuously throughout the experiment while keeping in phase with the stimulus. Occasionally the presented point-light actor stepped with the right foot over an obstacle as illustrated here. B, Schema of the experimental conditions. Eight periods of normal walking (white rectangles) were followed either by two periods either obstacle stepping (light-hashed rectangles) or six periods of arm swinging (dark-hashed rectangles) and ended with two periods of normal walking. Arrows indicate the time of electrical stimulation, occurring either during the changed visual stimulation (conditions: “with obstacle”, “standing/arm swing”), or during the 5th or 7th phase of normal walking (condition: “without obstacle”).

The stimuli were presented using a Macintosh computer running MotionViewer (version 48), an in-home programmed application (XCode 3.1 and OpenGL). The visual stimulus presented a slightly oblique back-view (facing to the right by 13°) which measured 11×5 cm on the 22″ TFT display that was connected to the iBook. To ensure that the observers perceived the stimulus as a back-view, the markers were occluded when covered by body parts. The back-view was chosen as the modulation of corticospinal excitability is maximal when the observed action corresponds to the orientation of the observer [19]. The TFT display was positioned at eye height, about 50–60 cm in front of the subject (Fig. 1A).

The point-light stimulus was based on 3-D recordings (Qualisys, Gothenburg, Sweden) of 16 points attached to a male actor (feet, ankles, knees, hips, wrists, elbows, shoulders and head). Three stimulus samples were thus obtained, each of which started with the phase corresponding to the right heel strike: (1) Walking on a treadmill at 3.5 km/h (0.97 m/s). (2) Walking and stepping with the right foot over an obstacle that moved with the surface of the (invisible) treadmill. The obstacle was displayed as a row of point-lights. (3) Standing still with the arms swinging as during normal walking [38]. In each of the three stimulus samples, the swing period of the arms was exactly matched. The stimulus samples 1 and 3 presented exactly one period (1.186 s), and that for the stepping over an obstacle (sample 2) had the duration of exactly two such periods. The stimulus sample 2 was recorded with an obstacle put on the surface of the treadmill, so that the actor stepped over the obstacle with the right foot. The three stimulus samples exactly matched the start and end postures, so that they could be repeatedly displayed in any order while appearing as a fluent continuous display.

Sixty-six trials were presented without interruption so the subjects were unaware of when a new trial started. Each trial started with eight periods of normal walking, and ended with two periods of normal walking. In between the initial and final walking periods either the stepping-over-an-obstacle stimulus or the arm-swing stimulus was presented, so each trial lasted 12/16 periods or 14.2/18.9 s (Fig. 1B). Movies of the two visual conditions are added as Movie S1 and Movie S2).

### Electrical stimulation

The application (MotionViewer) also triggered the electrical stimulation device (Digitimer DS7A, Welwyn Garden City, UK). The stimulation electrode (Axelgaard, Fallbrook, CA, USA) was placed at the medial side of right ankle, where the posterior tibial nerve is closest to the skin [39]. Trains of 8 biphasic rectangular pulses each of 2 ms duration at 200 Hz with a total duration of 40 ms were then applied with the constant-current stimulator. During quiet standing, the motor threshold was determined by gradually increasing the stimulus intensity until a visible muscle contraction was elicited in the *m. abductor hallucis*. The stimulation intensity was set on 1.5 times the motor threshold [40].

In each of the sixty-six trials, an electrical stimulation was applied at a time corresponding to 500 ms after heel strike of the right foot (Fig. 1B). In the obstacle condition the stimulation was applied in the stance phase of the right leg just before the subsequent swing phase during which the point-light actor would step over the obstacle (i.e. 500 ms after the visible obstacle appeared). In the arm-swing condition the stimulation was elicited in the second period of the stimulus standing still while swinging his arms. In the no obstacle condition the electrical stimulation was applied either in the 5th or the 7th period of normal walking. Each of these three conditions was applied twenty-two times in randomized order so the time of electric stimulation was unpredictable to the subjects.

### EMG and kinematic recordings

The EMG of the right TA muscle was recorded at 1000 Hz using bipolar, amplified surface electrodes (Biovision, Wehrheim, Germany). The recorded signal was rectified, band-pass filtered (30–300 Hz) and averaged using Matlab (Mathworks, Natick, MA, USA). To estimate the effect on the reflex responses, the rectified, band-pass filtered, averaged EMG responses were integrated over a time window of 60–140 ms from the onset of electrical stimulation for TA [8] and 100–200 ms from the onset of electrical stimulation for SOL, which is the time window that fully contained all subjects' reflex responses (Subject data are provided in Tables S1 and S2). No further post-hoc filtering or subtraction was applied. The difference in latency between both muscles is consistent with evidence suggesting that leg flexor and extensor muscles are controlled differentially in man and other animals [41]; for review see [42]. Also, cortico-spinal modulations as measured using sub-threshold MEPs have reported consistent stronger effects in the TA than in the SOL [43], [44].

**Figure 2:**
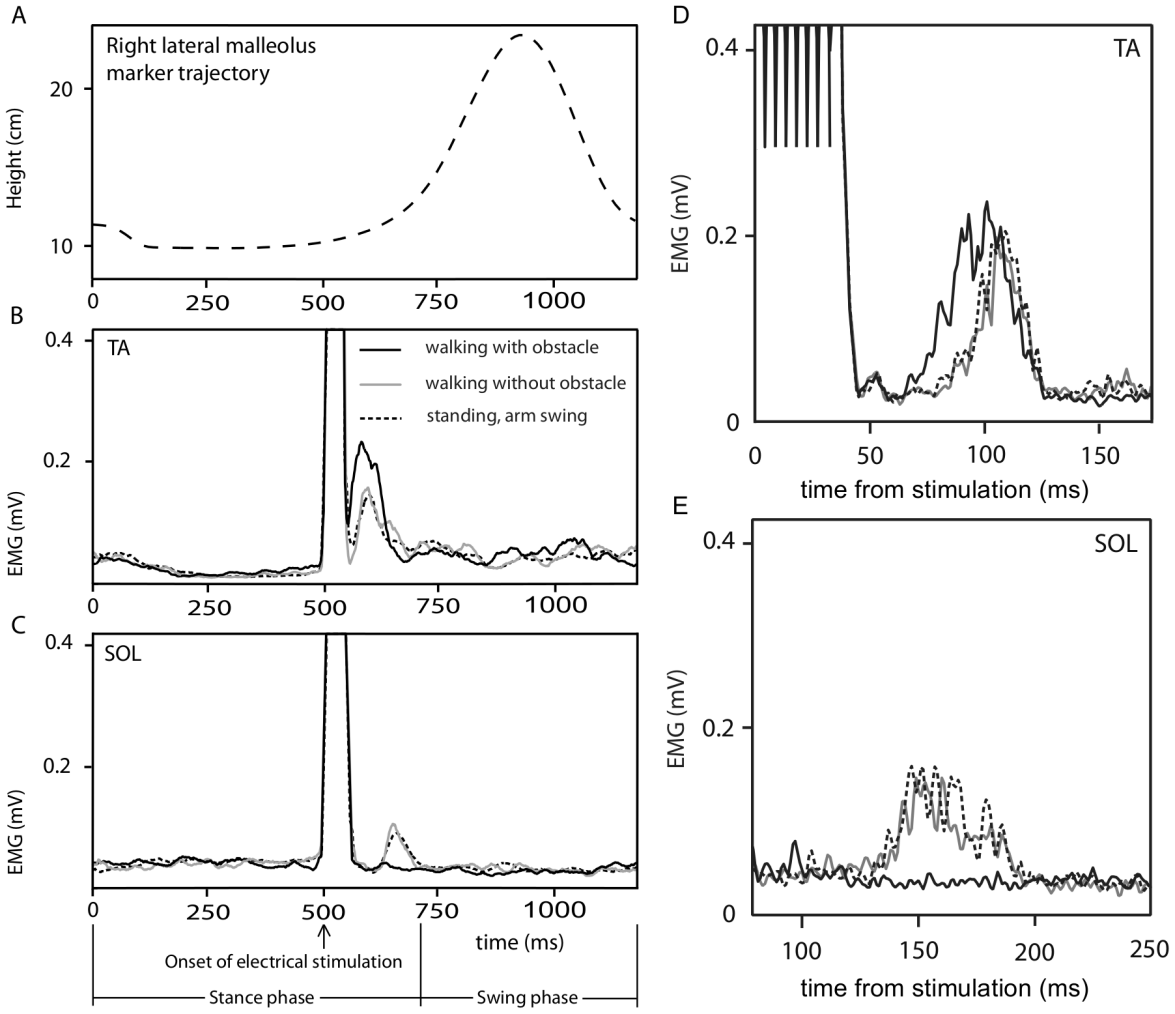
A–C, Mean full gait cycle of one subject. A, Height of the right ankle (lateral malleolus) averaged over all walking cycles. B–C, EMG signals of right *m. tibialis anterior*, TA (panel B) and right *m. soleus* (panel C) averaged over the gait cycles for each of the three conditions. Time t = 0 at right heel down. Onset of electrical stimulation was at t = 500 ms. D–E, Enlarged mean reflex responses of another subject. Continuous black traces: stimulation in the stance phase of the right leg during normal walking 500 ms after appearance of the obstacle - before obstacle avoidance step by the point-light-figure; grey traces: stimulation in the stance phase of the right leg during normal walking without a displayed obstacle; dashed black traces: stimulation during observed standing + arm swing. Stimulation was triggered in the same arm position as in the two other conditions.

Kinematic data of the right ankle (marker on the right lateral malleolus) of every participant was recorded (Qualisys, Gothenburg, Sweden) to compute the step height and step width of the right foot, to find out whether seeing someone stepping over an obstacle affected the kinematics of walking (Subject data are provided in Table S3). The EMG and kinematic data of one subject is shown in Figure 2A–C.

### Procedure

All participants could instantly describe the stimuli during a demonstration before the beginning of the experiment. Before the experiment, subjects practiced walking on the treadmill for about 10 min. During the second half of this practice period, the visual stimuli were displayed. The speed of the treadmill was adjusted until the subject's natural walking frequency matched the walking frequency of the presented point-light walker. The participants then practiced to keep on walking in phase with the observed point-light figure for several minutes. They were asked not to imitate the stepping movements nor the arm-swing movement, but simply to keep walking on while keeping in-phase with the stimulus. The participants were also instructed to keep on walking at the same frequency and to keep in phase with the observed arm-swing if the stimulus stood still and swung with its arms. All participants were able to follow these instructions.

During the main experiment, each of the three conditions was presented twenty-two times, in randomized order. The subsequent trials were presented continuously, without any discontinuity in between, so the participants could not discriminate when a new trial started. The main experiment lasted about 18 minutes.

### Analysis

The average reflex response data were subjected to repeated measures analyses of variance (ANOVAs) for the TA and the SOL. The within-factor Condition had levels WO (stimulus walking with approaching obstacle), ST (stimulus still standing with arms swinging), and NoO (stimulus walking without approaching obstacle). Planned post-hoc analyses (two-tailed) with Bonferroni-correction (N = 3) were used to analyze the hypotheses that responses TA and SOL are advanced and reduced respectively while observing a stepping movement as compared to the two control conditions.

From the kinematic data of the right ankle marker the step height and stride length were analyzed for walking cycles without electrical stimulation. Also, for these cycles without cutaneous stimulation, the average EMG responses were calculated. Repeated measures ANOVAs were performed on the step height, stride length, TA EMG, and SOL EMG, with planned post-hoc tests. Since for these measures we expected no difference, no Bonferroni correction was made.

## Results

Representative results of the reflex responses of two subjects in the three conditions and each recorded muscle are shown in Figure 2B–E. In both muscles, the response differed markedly when the stimulus presented stepping over an obstacle. These modulated responses to visually observed stepping were consistent with what is known for active stepping movements: the TA response was increased whereas the SOL response was absent.

**Figure 3:**
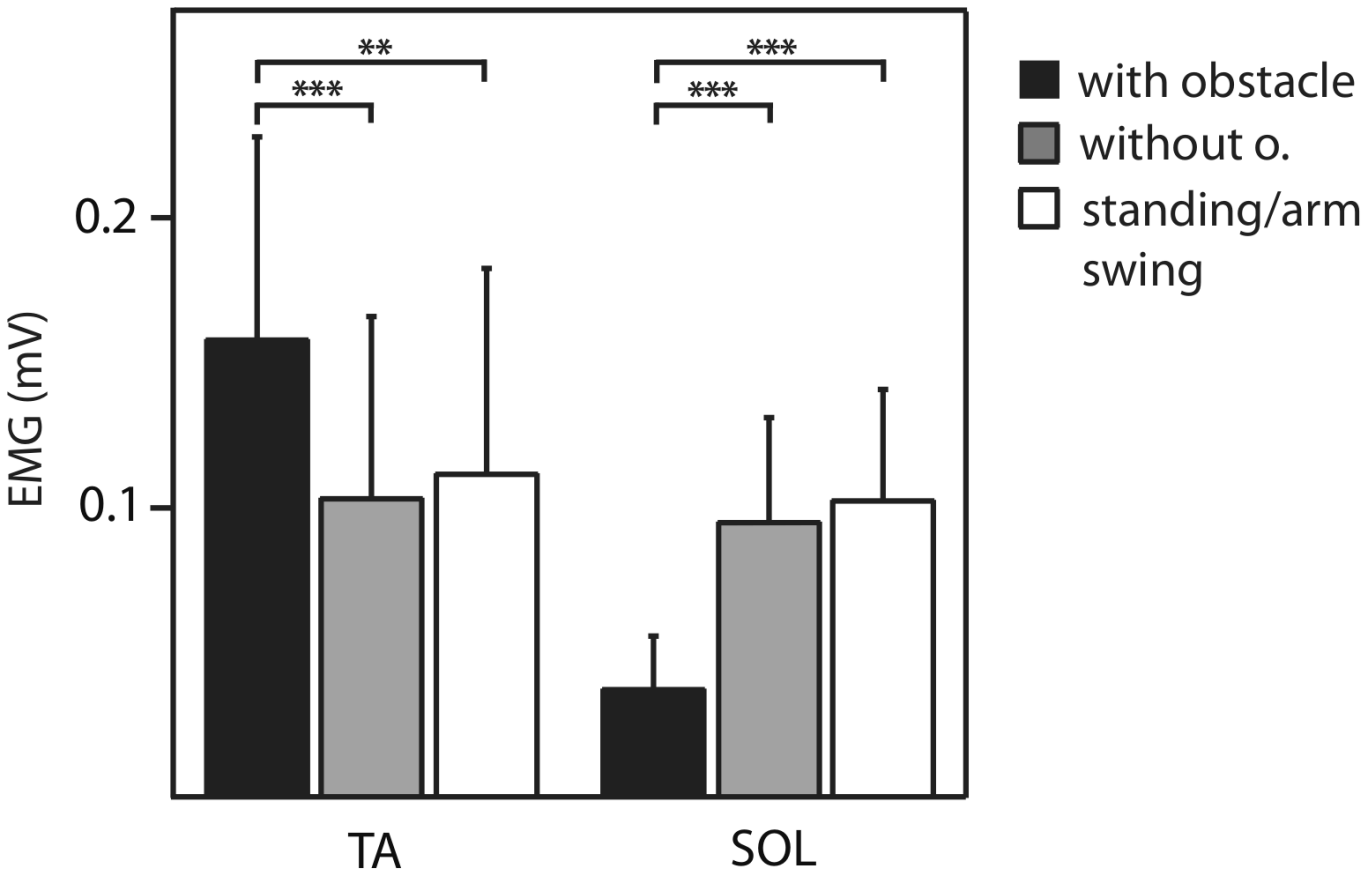
Mean reflex responses of ipsilateral *m. tibialis anterior* and ipsilateral *m. soleus* of all subjects (N = 8). Error bars represent one standard deviation.

The average data over all subjects are shown in Figure 3. The ANOVAs on the reflex data revealed that the main effects of Condition were statistically significant for TA (F_2,14_ = 10.3, p = 0.002) as well as for SOL (F_2,14_ = 17.9, p = 0.0001). For both muscles there were significant differences in the post-hoc tests between condition WO and both other conditions (Table 1).

**Table 1:** P-values of the post-hoc tests the three conditions in each muscle.

Conditions	TA p-values	SOL p-values
WO vs. ST	0.0033^**^	<0.0001^***^
WO vs. NoO	0.0008^***^	0.0002^***^
ST vs. NoO	0.4917^n.s.^	0.6004^n.s.^

Bonferroni-corrected significance levels: *p<0.0167, **p<0.0033, ***p<0.0017.

WO  =  Stimulus walking with approaching obstacle.

ST  =  Stimulus standing still with arms swinging.

NoO  =  Stimulus walking normally.

**Figure 4:**
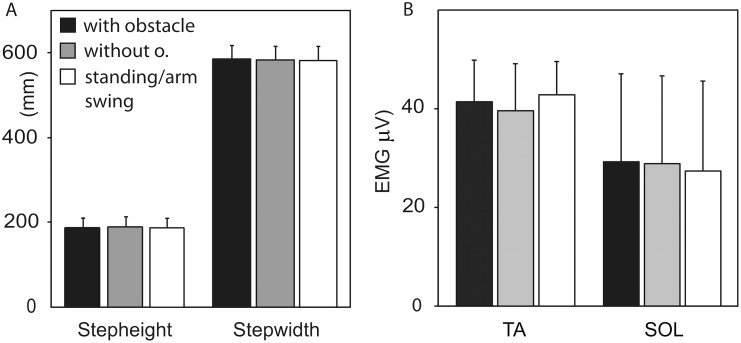
Mean kinematics and EMG data for strides without electrical stimulation. A, Average step height and step width (marker on right lateral malleolus) of all subjects in the conditions *with/without* approaching obstacle. Note that the obstacle step height of the point-light-walker exceeded the (visual) normal average step height by 60% (19% for the step width, respectively). B, Average EMG-responses for the three conditions computed over the same time windows as in Figure 3. Note the different scale as compared to Figure 3. Error bars represent one standard deviation.

The subjects were instructed to continue their normal walking pattern even if the stimulus made stepping movements or arm-swing movements. The kinematics and EMG patterns of walking cycles of one subject are shown in Figure 2A–C (note that the kinematics, panel A, for the different conditions aligned perfectly). Calculated over all all subjects, the average walking cycle lasted 1.186 s with a standard deviation of 4.5 ms. Average kinematic and EMG parameters for walking cycles without electrical cutaneous stimulation are shown in Figure 4. The kinematic and EMG patterns were indeed unaffected by the observed action as confirmed by the statistical results. In accordance with our hypothesis, neither the paired t-test on step height (t_7_ = 2.4, p = 0.12) nor on step width (t_7_ = 1.5, p = 0.22) could show significant differences.

## Discussion

The modulations in the reflex behavior of both, TA and SOL muscles were thus fully consistent with the modulations that are typically observed during active stepping over obstacles. The latter modulations are well established in the literature, e.g. [8]. These modulations are usually interpreted as preparatory behavior which probably includes an up-regulation of the spinal interneuronal TA-related circuits and down-regulation of SOL-related activity. Both responses are functionally useful as the TA dorsiflexes the foot and the suppressed activity of its antagonist plantar flexor SOL both assist obstacle avoidance when stepping over a sliding rod.

The results raise the question as to why the actual movements and EMG patterns are not modulated (as long as no reflex is evoked). Seeing an action, walking, while performing the same action simultaneously and in-phase is an easy everyday task. In our experiment, seeing a postural correction did not result in any measurable responses in the walking pattern of the participants of our study (cf. Fig. 4). This shows that the subjects were able to avoid automatic spontaneous imitation completely. We have proposed above two possible mechanisms by which the motor system might avoid such automatic imitative responses.

According to one possibility, the motor system would avoid automatic imitation by controlling the modulation due to visually perceived movement depending on ones own activity level. In that case, the modulation of responses should be strongly suppressed in the current experiment, because automatic imitation would have a strongly aversive effect on the postural stability of walking and would therefore increase the risk of falling. Depending on the level at which suppression occurs, two predictions can be derived for this mechanism. If the modulation is suppressed at a high level of control, observing the stepping over an obstacle would not affect the subject's walking pattern and should neither modulate the evoked muscular responses. Alternatively, if low-level suppression would occur, the whole motor command would be suppressed so that the walking pattern would be suppressed.

Instead, the recorded responses to cutaneous stimulation were modulated substantially. Importantly, the response in the ipsilateral anterior tibial muscle was enhanced (cf. Fig. 3), without having any influence on the actual movement kinematics in the absence of such cutaneous stimulation. Thus the results confirm that action observation does modulate neuronal networks for motor control down to peripheral levels during walking [12]. Moreover, the results support the alternative hypothesis, i.e., that the high-level motor commands are modulated by the observed perturbation, without any suppression of the descending commands.

Observing the arm-swing movement during active walking did not modulate the evoked responses to cutaneous stimulation. Although a null-result is not a strong finding, we still think it is potentially interesting. It is known that evoked postural responses are different in walking and standing still with the arms swinging [38]. Thus in our results, the observation of a corrective response to the action (walking) performed by the subjects affected the evoked responses whereas the observation of a different but similar action did not. A number of explanations can be given for the absence of an effect in the arm-swing condition. (1) The effect might have been too small to measure in the current set-up, (2) modulations to corrective responses only occur if they fit the potential repertoire of responses that match the motor context of the current action, or (3) modulations only occur if they represent an enhanced activity. Further experiments are needed to clarify this result.

There is electrophysiological evidence that primary motor cortex neurons exhibit a mirror-like activity during action observation [45]–[47] and it was shown that some neurons of the entire pool of recorded pyramidal tract neurons increased their discharge while others were suppressed during action observation [47]. Conversely, the facilitated neurons exhibited a decreased activity and the suppression-type neurons became active during action execution. The authors inferred that thereby a direct input to the spinal circuits is partly or completely reduced during action observation, but this would not explain why in our experiment the participants' unperturbed walking was in no way affected by the observation of occasional stepping movements.

Our finding thus has an important consequence for the cortical control of movements, for it implies that there is a population of motoneurons in the brain that actively controls movements without directly evoking any muscular responses during an ongoing action, as long as the action is not perturbed. Thus, the mirror properties of pyramidal tract neurons do not reflect some sort of passive “motor resonance behavior”, but rather reflect highly adaptive preparatory motor response to a perturbation that might be occurring. Such a function is essential for fast, dynamic, task-specific postural responses and provides a cue to the underlying mechanism to such task-specific reflexes. This is highly adaptive, for in everyday life the stumbling or stepping movement made by a person walking directly in front of us is an important cue for a possible obstacle on our way.

It is not a novel idea that motor simulation is predictive. Monkey mirror neurons were found to become facilitated when the final part of a hand action could not be seen but therefore only be inferred [48]. In humans, the mere knowledge of an upcoming movement was sufficient to automatically activate the observers motor system which was interpreted as a process of generating a prediction of another person's movement [49]. An activation of the motor system using TMS was also found in case an incomplete sequence of static snapshots showing the initial phases of grasp or flick actions was presented [50].

These findings including the present study support the notion that an overt facilitation of the motor system during action observation on the one hand represents a currently observed movement and on the other hand represents the process of generating a predictive, internal model of the anticipated action [51]. This process of mentally simulating motor actions during its perception as a way to verify prior predictions about intentions or goals was termed *Predictive Coding* [52], [53].

## Conclusions

1. It is known that cutaneously evoked reflexes in the anterior tibial and soleus muscles are modulated by the active execution of an action such as stepping over an obstacle, and also by the passive observation while sitting quietly. We found that viewing occasional stepping over an obstacle during active walking also modulates evoked reflexes.
2. The reflexes are modulated as if the walking participant is actively performing a stepping movement, rather than merely observing one.
3. The walking movements were unaffected by the observed stepping movements.
4. This indicates that the observed actions did not affect the ongoing motor patterns but rather modified the preparatory activity to action pattern repertoires that might come up in the near future. We conclude that spinal excitability during action observation is not an adverse side-effect of action understanding but reflects adaptive and predictive motor control.

## Supporting Information

Table S1 TA iEMG.
(TEX)

Table S2 SOL iEMG.
(TEX)

Table S3 Kinematic data.
(TEX)

Movie S1 **Movie shows the conditions with six periods of arm swing after eight periods of treadmill walking.**
(MOV)

Movie S2 **Movie shows the conditions with obstacle stepping.**
(MOV)
